# Enterogenous Microbiotic Markers in the Differential Diagnosis of Crohn’s Disease and Intestinal Tuberculosis

**DOI:** 10.3389/fimmu.2022.820891

**Published:** 2022-03-16

**Authors:** Mingshan Jiang, Zhen Zeng, Kexin Chen, Yuan Dang, Lili Li, Chunxiang Ma, Rui Cheng, Kehan Hu, Xi Li, Hu Zhang

**Affiliations:** ^1^ Department of Gastroenterology, West China Hospital, Sichuan University, Chengdu, China; ^2^ Centre for Inflammatory Bowel Disease, West China Hospital, Sichuan University, Chengdu, China; ^3^ Laboratory of Inflammatory Bowel Disease, Frontiers Science Center for Disease-Related Molecular Network, West China Hospital, Sichuan University, Chengdu, China

**Keywords:** Crohn’s disease, enterogeneous microbiotic markers, intestinal tuberculosis, diagnosis, differential diagnosis

## Abstract

Crohn’s disease (CD) is a chronic intestinal disorder characterized by refractory gastrointestinal ulcerations. Intestinal tuberculosis (ITB) is one common intestinal disease in east Asia. The two diseases share similar clinical manifestations and endoscopic characteristics. Thus, it is difficult to establish a definite diagnosis of CD, CD concomitant with ITB (CD-ITB), and ITB in practice. Some enterogeneous microbiotic markers have been applied to differentiate CD and ITB, but it remains unknown how they work for the three groups of patients. The aim of our study was to explore the diagnostic values of these enterogeneous microbiotic markers (ASCA IgG, ASCA IgA, ACCA, Anti-I2 and AMCA) among CD, CD-ITB, and ITB patients. A total of 124 individuals were retrospectively enrolled in this study, namely, 103 CD patients, 10 CD-ITB patients, 9 ITB patients, and 68 healthy controls. The demographic and clinical characteristics of these patients were collected and analyzed. The values of these individual or combined enterogeneous microbiotic markers in diagnosis and classification were assessed in CD, CD-ITB, and ITB patients. ASCA IgG, ASCA IgA, and AMCA could accurately differentiate CD patients from healthy controls with an area under curve (AUC) of 0.688, 0.601, and 0.638, respectively. ASCA IgG was significantly higher in CD patients than in CD-ITB patients (P = 0.0003). The Anti-I2 antibody was appropriate for distinguishing CD-ITB from ITB patients (P = 0.039). In CD patients, ASCA IgG was higher in severe patients than in mild (P <0.0001) and inactive patients (P <0.0001), respectively. AMCA was significantly elevated in severe and moderate patients compared to inactive patients (P = 0.001, P = 0.003, respectively). AMCA was associated with a higher risk of CD-related surgery with a significant P-value of 0.0038. In our cohort, ASCAs and AMCA could accurately distinguish CD from healthy controls with an acceptable AUC. A combination of elevated ASCA IgG and AMCA antibodies established a higher sensitivity in differentiating CD from healthy controls. Elevated ASCA IgG demonstrated a differential diagnostic value between CD and CD-ITB. Anti-I2 could also distinguish CD-ITB from ITB. The level of AMCA was associated with both disease severity and CD-related surgery. Likewise, the level of ASCA IgG was also related to disease severity.

## Introduction

Crohn’s disease (CD) is a chronic intestinal inflammatory disease with remittent and progressive inflammation (1). Intestinal tuberculosis (ITB) is a second type of chronic intestinal disorder. Both CD and ITB share similar clinical manifestations such as intermittent abdominal pain and diarrhea. In addition, both of them are characterized by gastrointestinal ulcerations. Therapeutic medications such as immunosuppressants, glucocorticoids, or even biologics are recommended for CD, but they are contraindicated in ITB, because these medications can definitely aggravate ITB. Therefore, it is of great importance to make a differential diagnosis between CD and ITB before embarking on treatment.

However, it is a substantial challenge to differentiate CD from ITB in practice. Firstly, CD does not have a golden diagnostic criterium. Its detailed pathogenesis still remains unknown. Recent studies have suggested that CD results from an interaction among genetic susceptibility, commensal microorganism dysbiosis, and dysregulated immune responses (2). Its diagnosis and classification is based on the combination of clinical manifestations, laboratory data, endoscopy characteristics, radiology and histopathology (3). Secondly, ITB is caused by a tuberculous infection. In theory, a definite diagnosis of ITB can be made if the presence of *Mycobacterium tuberculosis* (TB) is confirmed by Polymerase Chain Reaction (PCR) or fast acid staining. Unfortunately, it is still very difficult or very rare to find TB in biopsies from patients with ITB. Thus, the diagnosis of ITB is still based on a combined evaluation of clinical, endoscopic, histologic, and radiographic findings. Thirdly, more importantly, some patients can be simultaneously affected by both CD and ITB, or affected consecutively, which makes it more difficult to make a precise differential diagnosis between CD, CD-ITB, and ITB.

Endoscopy is a critical tool to detect intestinal lesions such as erosion, ulcerations, fistulous orifices, and stricture in CD and ITB patients. Although it plays a critical role in diagnosis and differential diagnosis, it is invasive, expensive and time-consumption, making patients refuse endoscopy.

Many studies have been undertaken to explore enterogeneous microbiotic markers which can contribute to a valid differential diagnosis. Enterogenous microbiotic markers in the blood are specific serum antibodies activated by luminal antigens such as gut microbiota and food antigens (4, 5). A growing body of evidence has demonstrated that serological surrogates are additional tools for diagnosis and classification. To date, the most promising biomarkers contain anti-*Saccharomyces cerevisiae* (ASCA, the antibody triggered by the mannan in the cell wall of *S. cerevisiae*), anti-outer membrane protein C (anti-OmpC, the antibody triggered by the outer membrane porin C transport protein of *Escherichia coli*), anti-mannobioside carbohydrate IgG antibodies (AMCA), anti-chitobioside carbohydrate IgA (ACCA), anti-laminaribioside carbohydrate IgG antibodies (ALCA), and Anti-I2 antibody (the antibody triggered by *Pseudomonas fluorescens* component I2) (6–8). Fecal biomarkers, such as calprotectin and lactoferrin have also been used in the diagnosis and classification of diseases (6, 9). These biomarkers have their own specific advantages in distinguishing CD from other types of colitis, predicting disease-related surgery and assessing the risk of disease relapse (10–13). Nevertheless, few studies have estimated the expression levels of these serologic biomarkers in CD-ITB patients. To date, however, a noninvasive gold standard test for the differential diagnosis between CD, CD-ITB, and ITB has not been developed yet.

Hence, it is critical to investigate whether these microbiotic markers could be used in the diagnosis of CD, CD-ITB, and ITB, and whether these biomarkers are associated with disease severity or phenotype. In this study, we aimed to explore the expression levels of ASCA IgG, ASCA IgA, ACCA, Anti-I2, and AMCA in CD, ITB, and CD-ITB patients, and to evaluate the value of these serologic biomarkers in diagnosis, differential diagnosis, prediction of phenotype and assessment of disease severity. Our study found that some specific or a combination of enterogeneous microbiotic markers can contribute to a better differential diagnostic value between CD-ITB and CD, CD-ITB and ITB.

## Materials and Methods

### Patients and Study Characteristics

A total of 124 patients were retrospectively enrolled in this study, namely, 103 patients with CD, 10 patients with both CD and ITB, 9 patients with ITB and 68 healthy controls from the Department of Gastroenterology of the West China Hospital between January 2015 and February 2019 The diagnosis of CD was based on clinical history and examination, endoscopy, small bowel imaging, radiological image (CT, MRI or ultrasound), blood tests and histological evidences which based on ECCO-ESGAR Guideline (13). The diagnosis for ITB should meet either one of the following criteria: 1) the presence of acid-fast bacilli or caseating granuloma in pathological specimens, 2) the presence of *mycobacterial tuberculosis* in tissue culture, 3) positive TB DNA-PCR in mucosal biopsies, 4) effective response to anti-tuberculosis treatment (13). The diagnosis of CD-ITB should be content with both CD and ITB criteria. Healthy controls were volunteer individuals with no IBD and no gastrointestinal disorder. Clinical manifestations, physical examinations, and laboratory data were recorded based on the Crohn’s Disease Activity Index (CDAI) criteria. Endoscopy characteristics, radiology image, histopathology and treatment strategies were also systematically recorded in this study. Disease activity was determined by Best CDAI score. CDAI <150 was defined “inactive”, CDAI between 150 and 220 was defined “mild”, CDAI between 220 and 450 was defined “moderate”, CDAI >450 was defined “severe” respectively. Clinical phenotypes of CD patients were determined based on the Montreal Classification (14). This study was reviewed and approved by the ethical committee of the West China Hospital.

### Enterogenous Microbiotic Marker Analysis

All the serum samples from patients and healthy controls were stored at −80°C refrigerator in the laboratory until analysis. All serum samples were analyzed by enzyme-linked immunosorbent assay (ELISA).

### Statistical Analysis

Statistical analysis was performed with SPSS 22.0 statistical software package and GraphpadPrism 9.0 statistical software package. The Student’s t-test, Mann–Whitney, Kruskal–Wallis, and Logistic regression tests were used as appropriate. Receiver operating characteristic (ROC) curve was analyzed for the definition of the cut-off values and the assessment of the diagnostic accuracy. A P-value <0.05 was considered significant.

## Results

### Demographic Data and Clinical Characteristics

Overall, 103 CD patients (72 men, median age 28.7 years), 9 ITB (7 men, median age 44.2 years) patients, 10 CD-ITB (5 men, median age 28.9 years) patients and 68 (40 men, median age 31.1 years) healthy controls were retrospectively enrolled in this study. A majority of CD and CD-ITB patients were diagnosed at ages 17–40. In this study, 94 (92.3%) CD patients and all the CD-ITB patients were contented with the criteria for active disease. Of the 103 CD patients, 35(34%) represented ileocolonic lesion, 28 (27.2%) represented colonic lesion, 27 (28.2%) represented small bowel lesions, 8 (7.8%) represented terminal ileal lesion, and 3 (2.9%) represented upper gastrointestinal lesion. Of the 10 CD-ITB patients, 7 (70%) represented ileocolic lesion, 2 (25%) represented colonic lesion, 1 (10%) represented upper gastrointestinal lesion, and 1 (10%) represented small bowel lesions. Of the 9 ITB patients, 7 (77.78%) represented colonic lesion, 1 (11.11%) represented ileocolonic lesion, and 1 (11.11%) represented upper gastrointestinal lesion. According to the Montreal classification, 48 (46.6%) CD patients had a complicated behavior of structuring or penetrating, 28 (27.2%) patients had perianal disease. Almost one third CD-ITB patients had structuring or penetrating disease behavior and half of the patients had perianal disease. Almost half of both CD and CD-ITB patients suffered from surgery in the course of disease. Demographic data and baseline disease characteristics of CD patients, CD-ITB patients, ITB patients, and healthy controls are listed in **Tables 1** and **2**.

**Table 1:** Demographic and main baseline characteristics of CD patients, CD combined ITB and controls.

Characteristics	CD (n = 103)	CD-ITB (n = 10)	Controls (n = 68)
**Male, n (%)**	72 (69.9%)	5 (50%)	40 (58.8%)
**Median age at diagnosis**	28.7 (14–64)	28.9 (14–51)	
≤16 years [A1], n (%)	9 (8.7%)	1 (10%)	
17–40 years [A2], n (%)	77 (74.8%)	7 (70%)	
≥40 years [A3], n (%)	17 (16.5%)	2 (20%)	
**Smoking status**			
Current, n (%)	29 (28.2%)	2 (20%)	
Never, n (%)	20 (19.4%)	2 (20%)	
Ex-smoker, n (%)	54 (52.4%)	6 (60%)	
**Disease activity**			
Active, n (%)	94 (92.3%)	10 (100%)	
Mild, n (%)	33 (32%)	2 (20%)	
Moderate, n (%)	41 (39.8%)	4 (40%)	
Sever, n (%)	20 (19.4%)	4 (40%)	
Inactive, n (%)	9 (8.7%)	0	
**Surgery, n (%)**	44 (42.7%)	4 (40%)	
**Disease location**			
Terminal Ileal [L1], n (%)	8 (7.8%)	0	
Colonic [L2], n (%)	28 (27.2%)	2 (25%)	
Ileocolonic [L3], n (%)	35 (34%)	7 (70%)	
Upper gastrointestinal [L4], n (%)	3 (2.9%)	1 (10%)	
Small bowel, n (%)	27 (28.2%)	1 (10%)	
Small bowel with Ileocolonic, n (%)	9 (8.7%)	0	
Small bowel with Terminal Ileal, n (%)	3 (2.9%)	0	
Upper gastrointestinal with Ileocolonic, n (%)	3 (2.9%)	1 (10%)	
**Disease behavior**			
Non-stricturing, non-penetrating [B1], n (%)	55 (53.4%)	6 (60%)	
Stricturing [B2], n (%)	34 (33%)	3 (30%)	
Penetrating [B3], n (%)	14 (13.6%)	1 (10%)	
Perianal disease, n (%)	28 (27.2%)	5 (50%)	
Non-stricturing, non-penetrating with Perianal disease, n (%)	18 (17.5%)	2 (20%)	
Stricturing with Perianal disease, n (%)	6 (5.8%)	2 (20%)	
Penetrating with Perianal disease, n (%)	1 (1%)	0	

**Table 2:** Demographic and main baseline characteristics of ITB patients.

Characteristics	ITB (n = 9)
**Male, n (%)**	7 (69.9%)
**Median age at diagnosis**	44.22 (23–70)
**Smoking status**	
Current, n (%)	7 (77.78%)
Never, n (%)	1 (11.11%)
Ex-smoker, n (%)	1 (11.11%))
**Disease location**	
Terminal Ileal, n (%)	0
Colonic, n (%)	7 (77.78%)
Ileocolonic, n (%)	1 (11.11%)
Upper gastrointestinal, n (%)	1 (11.11%)
**Disease behavior**	
Non-stricturing, non-penetrating, n (%)	9 (100%)
Stricturing, n (%)	0
Penetrating, n (%)	0
Perianal disease, n (%)	0

### Serologic Markers Reactivity in CD, ITB, and Control Groups

**Figure 1:**
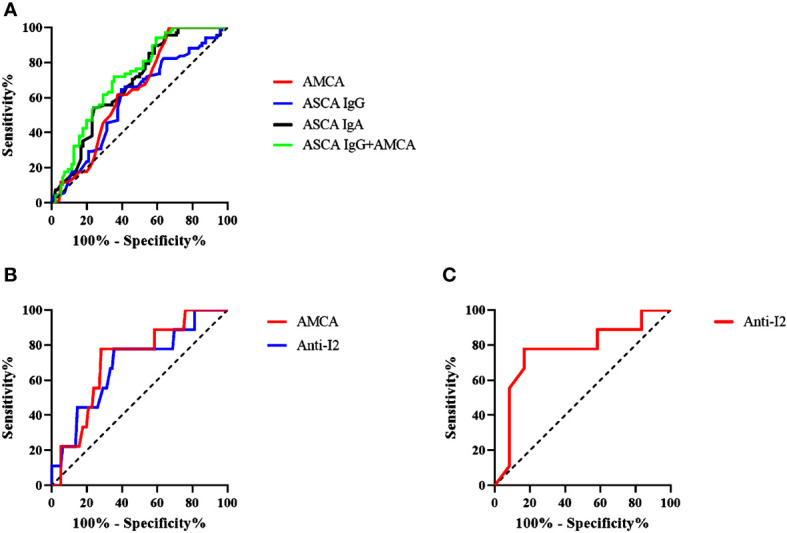
**(A)** Receiver-operating characteristics (ROC) analysis of the discrimination power of ASCA (IgA and IgG), AMCA and ASCA IgG combined with AMCA in patients with CD (n = 103) and healthy control (n = 68); **(B)** ROC analysis of the discrimination power AMCA and Anti-12 in patients. **(C)** ROC analysis of the discrimination power of Anti-I2 in patients with CD-ITB (n=10) and ITB (n=9).

ROC analysis was utilized to evaluate the diagnostic values of ASCA IgG, ASCA IgA, AMCA, ACCA, and Anti-I2 in CD. For differentiating CD patients from healthy controls, the AUCs were 0.688, 0.601, and 0.638 for ASCA IgG, ASCA IgA, and AMCA respectively. The cut-off parameters of ASCA IgG and AMCA were 24.65 and 80. ASCA IgG performed better than the other 4 biomarkers in distinguishing CD patients from healthy controls (**Figure 1A**). The combination of ASCA IgG and AMCA improved the diagnostic value with an AUC of 0.72, a sensitivity of 65% and a specificity of 72.1% (**Figure 1A**). For differentiating CD from ITB, AMCA and Anti-I2 demonstrated the most valuable AUC of 0.712 and 0.691, with the sensitivity of 71.8%and 64.1%, specificity of 77.8 and 77.8%, respectively (**Figure 1B**). The cut-off parameters of these two antibodies were 45.5 and 0.419.

### Association Between Biomarkers and Disease Severity in CD Patients

**Figure 2:**
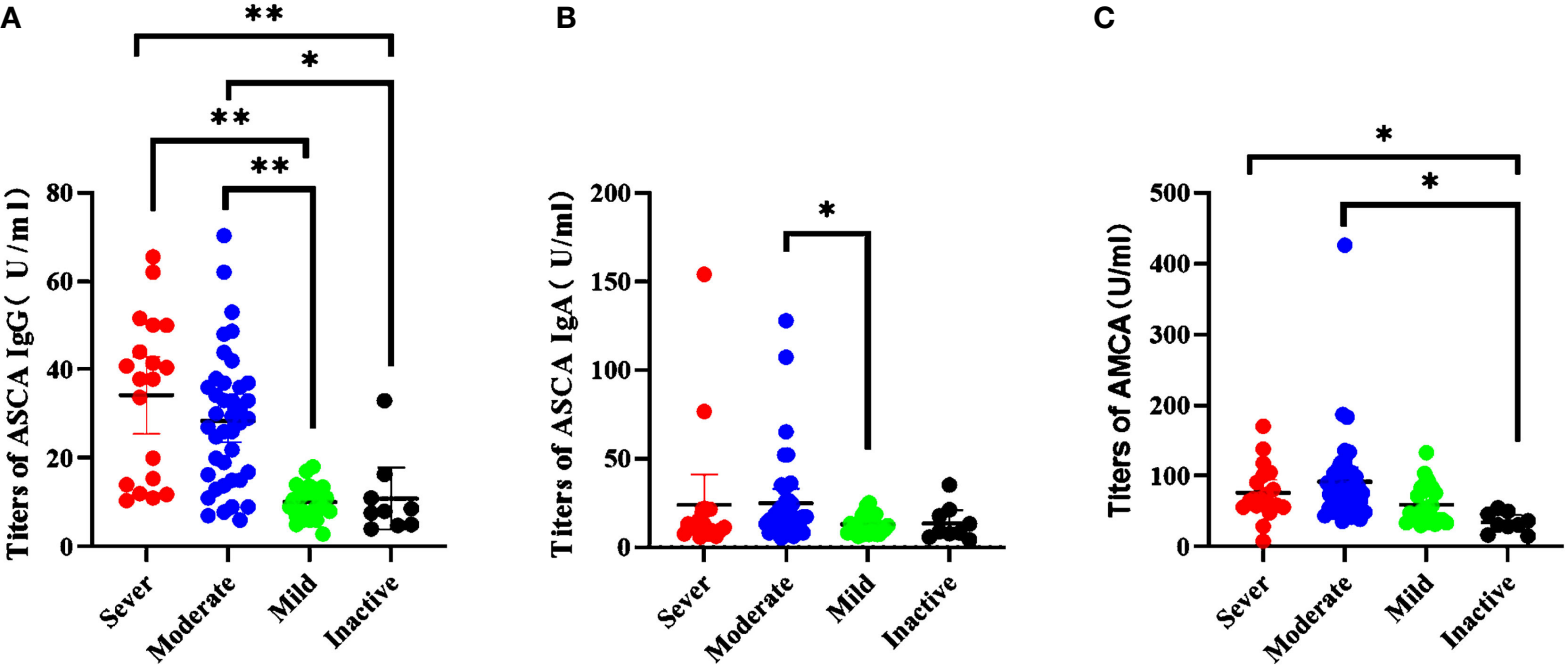
Association between serological markers and the severity during the disease course in CD patients. **(A)** The titers of ASCA IgG in disease severity *P = 0.002, **P < 0.0001); **(B)** The titers of ASCA IgG in disease severity (P = 0.031); **(C)** The titers of AMCA in disease severity (sever vs inactive, P = 0.003; moderate vs inactive, P = 0.001).

We investigated the association between biomarkers and disease severity in CD patients. Based on CDAI scores, 9 (8.7%) CD patients were defined “inactive”, 33 (32%) were defined “mild”, 41 (39.8%) were defined “moderate”, and 20 (19.4%) were defined “severe”. Kruskal–Wallis analysis was performed to assess whether the biomarkers were associated with disease severity. A significant higher proportion of patients with severe disease were ASCA IgG positive patients as compared to mild patients (P <0.0001) and inactive patients (P <0.0001). The same results were also established in moderate patients as compared to mild patients (P <0.0001) and inactive patients (P = 0.002; **Figure 2A**). However, the level of ASCA IgG showed no difference between severe patients and moderate patients. ASCA IgA antibody only have difference between mild patients and moderate patients (P = 0.031; **Figure 2B**). AMCA was significantly elevated in severe and moderate patients compared to inactive patients (P = 0.001, P = 0.003; **Figure 2C**). In contrast, ACCA and Anti-I2 showed no significant differences in disease severity.

### Association Between Biomarkers and Disease Phenotype in CD Patients

**Figure 3:**
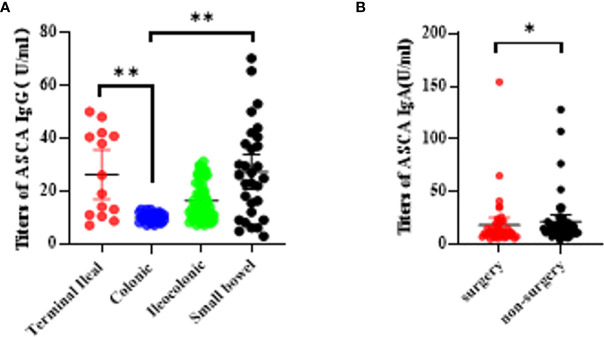
Association betweeen serological markers and the disease phenotype during the disease course in CD patients. **(A)** The titers of ASCA IgG in disease location (*P < 0.05, **P < 0.0001); **(B)** The titers of AMCA in disease related surgery (P = 0.0038).

We further explored the relationship between biomarkers and disease locations. We found that only ASCA IgG was statistically related to an increased risk of terminal ileal and small bowel lesion compared to colonic lesion (P <0.0001; **Figure 3A**). Unfortunately, we failed to detect significant associations between any other biomarkers and disease locations. With regard to disease behaviors, we also failed to find significant differences in these biomarkers. Besides, we further evaluated the relationship between the biomarkers and the CD-related surgery. Among all the five biomarkers, only AMCA antibody was associated with a higher risk of CD-related surgery with a significant P-value of 0.0038 (**Figure 3B**).

### Distinguishing CD-ITB From ITB Patients, CD-ITB From CD Patients, CD-ITB Patients From Healthy Controls

**Figure 4:**
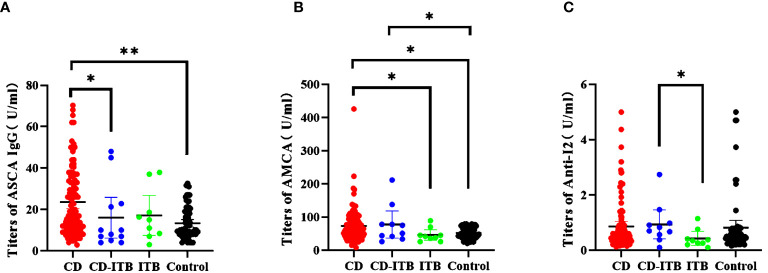
Distinguishing CD combined ITB patients from ITB patients, CD patients and healthy controls.**(A)** The titers of ASCA IgG (CD vs CD-ITB P = 0.0351; CD vs Control P < 0.0001; **(B)** The titers of AMCA (CD vs ITB, P = 0.0341; CD vs Control, P = 0.00222; CD-ITB vs ITB, P = 0.071;CD-ITB vs Control P = 0.0022); **(C)** The titers of Anti-I2 (CD-ITB vs ITB, P = 0.044; CD-ITB vs Control, P = 0.0553) *P < 0.05, **P < 0.0001.

We finally investigated the expression levels of these markers in CD-ITB patients. The level of ASCA IgG was significantly higher in CD patients than in CD-ITB patients (P = 0.048, **Figure 4A**). However, ASCA IgG expressed no difference between CD-ITB and ITB patients, CD and ITB patients. On the contrary, AMCA was notably associated with CD patients compared to ITB patients (P = 0.0341, **Figure 4B**). The level of AMCA showed no difference between CD and CD-ITB patients, CD-ITB and ITB patients (P = 0.071). For CD-ITB, we found that ASCA IgG antibody was the best serological marker to distinguish CD from CD-ITB patients (AUC = 0.655). In addition, Anti-I2 was the most appropriate biomarkers for distinguishing CD-ITB from ITB patients (Anti-I2, AUC = 0.767; AMCA, AUC = 0.683, **Figure 1C**). AMCA were also suitable for distinguishing CD-ITB patients from healthy controls with an AUC 0.668. Compared to the ITB patients, Anti-I2 were significantly higher in the CD-ITB patients (P=0.044, **Figure 4C**).

## Discussion

In this study, we included a unique cohort of 103 CD individuals, 10 CD-ITB individuals, 9 isolated ITB individuals and 68 healthy controls. All five biomarkers (ASCA IgG, ASCA IgA, AMCA, ACCA and Anti-I2) were analyzed. Their diagnostic values were assessed in this well-defined Chinese cohort. We specifically identified a diagnostic role of ASCA IgG, Anti-I2 and AMCA in distinguishing CD from CD-ITB, CD from healthy controls and CD-ITB from ITB patients. In addition, the associations between biomarkers and disease behavior and also disease severity were also investigated.

Based on our results, the levels of ASCA IgG, AMCA, and Anti-I2 had the highest diagnostic value. Our data suggested that ASCA IgG antibody titers were significantly higher in CD patients than in CD-ITB patients and healthy controls. AMCA antibody titers were significantly higher in CD patients than ITB patients and healthy controls. A previous study reported the similar results that both ASCA IgA and IgG antibodies have a high specificity for patients with CD. ASCA was also confirmed to have the highest value of differentiating CD patients from healthy controls according to a meta-analysis including fourteen studies (15). Both ASCA IgA and IgG antibodies were positive in 39–70% of CD patients and 20–25% of healthy relatives (16, 17). Moreover, our study demonstrated that the levels of ASCA IgG and AMCA showed significant differences in the subgroups based on disease severity in the CD patients. According to our search, ASCA IgG and AMCA were significantly higher in both severe and moderate subgroups of patients. Regarding the disease locations, patients with small bowel lesion and terminal ileal lesion had the highest levels of ASCA IgG. AMCA and ACCA exhibited no differences in any of the subgroups based on disease locations. However, a study from Malickova et al. demonstrated that AMCA was related to small intestinal lesion in CD patients (18). Another study suggested that anti-glycan ALCA was significantly associated with an increased risk of colonic or ileocolonic lesion (19). Furthermore, a study of 1,225 IBD patients indicated that in CD patients, both ASCA and AMCA were related to a poor prognosis such as complicated behaviors (strictures or fistulas), ileal involvement, and the necessary for abdominal surgery (20). One study from Kaul et al. indicated that ACCA is the most valuable serological biomarker to be associated with complications. Not only ASCA but also ACCA was related to the need of surgery (19). However, another study has illustrated that a combination of pANCA (perinuclear antineutrophil cytoplasmic antibody, the antibody triggered by granules of neutrophil cytoplasm) and ASCA demonstrated encouraging diagnostic values that estimated a specificity of approximately 90% for CD (21). In addition, Vasiliauskas et al. revealed that CD patients with elevated ASCA and descended pANCA were associated with 100% advanced fibrostenosis, 79% internal penetrating complications and 86% bowel surgery (22). In contrast to those studies, our data indicated that ASCA IgG and ACCA showed no differences in CD-related surgery nor penetrating nor structuring complications. AMCA had the highest association with CD-related surgery. No correlation was found between the level of AMCA and small intestinal lesion in CD. Our study also illustrated that the combination of ASCA IgG and AMCA demonstrated encouraging diagnostic values with a sensitivity of 65% and specificity of 72.1%, which may provide a selectable method to differentiate CD patients from healthy controls. Therefore, ASCA IgG and AMCA could be recommended as a biomarker for some clinical features of CD. After all, our results confirmed the values of ASCA and AMCA in diagnosis, differential diagnosis, prediction of phenotype and assessment of disease severity of CD.

In most cases, the definite diagnosis of CD and its differential diagnosis from ITB could be made based on a combinational analysis of the clinical manifestations, endoscopy characteristics, medical histology, radiology and histopathology results. However, the diagnosis of CD, particularly CD-ITB, can be misled or delayed due to the similar characteristics. It is difficult to make an accurate diagnosis of tuberculosis infection in the intestine through histology or PCR technique due to their high false negative rates. It is even more challengeable to define CD-ITB diagnosis through histopathological features. Almost all of the existing studies have demonstrated that clinical variables, such as clinical symptoms, radiologic parameters and endoscopic characteristics were helpful to differentiate CD from ITB (15, 23–27). Given these challenges, it is crucial to develop some accurate noninvasive diagnostic biomarkers to pinpoint CD-ITB and to differentiate this disorder from other intestinal diseases. The ideal noninvasive diagnostic test must exhibit a high sensitivity and specificity. Unfortunately, although great advances have been made in experimental methods, no biomarker-associated methods have been reported yet to achieve this aim. A large number of studies have assessed ASCA, ACCAs, AMCA, Anti-I2 and pANCA as diagnostic markers in UC, CD and non-IBD patients. Due to the low specificity, these biomarkers showed a limited value in differential diagnosis. A prospective study which enrolled 40 CD patients and 40 ITB patients indicated that positive ASCA IgG and ASCA IgA antibodies were independent markers for differentiating CD from ITB (28). The diagnostic values of these biomarkers have not been studied in CD-ITB patients yet. It should be noted that both ASCA IgG and ASCA IgA were lower in CD-ITB than in CD in our study. More interestingly, the levels of ASCA IgG and ASCA IgA showed no difference between CD-ITB and healthy controls.

Altogether, our study suggests that Anti-I2 antibody could be a sensitive and specific tool to distinguish CD-ITB from CD patients. Compared to the ITB patients, Anti-I2 was significantly higher in the CD-ITB patients. Anti-I2 also displayed a better discriminatory capability over ASCA IgG in differentiating CD-ITB from ITB patients. Furthermore, AMCA showed no difference between CD-ITB and CD patients. AMCA was higher in CD-ITB patients than in ITB patients. Overall, our study suggests that CD-ITB patients might have normal ASCA IgG and ASCA IgA levels and higher AMCA and Anti-I2 levels. AMCA and Anti-I2 in ITB patients showed a trend for being negative.

The present retrospective study clearly highlighted the value of biomarkers in the setting of disease diagnosis between CD, CD-ITB, ITB patients and healthy controls. Nevertheless, our research has some limitations: Firstly, this was a retrospective study. Secondly, some cohorts have a small group of patients, the number of CD-ITB and ITB patients in this study limited the conclusions of the utility of these biomarkers. Finally, a longitudinal prospective study within a large number of patients must be performed to assess those findings.

In conclusion, ASCAs and AMCA could all be used to distinguish CD from healthy controls. A combination of elevated ASCA IgG and AMCA antibodies established a higher sensitivity in differentiating CD from healthy controls. Elevated ASCA IgG demonstrated a differential diagnostic value between CD and CD-ITB. Anti-I2 could also distinguish CD-ITB from ITB. The level of AMCA was associated with both disease severity and CD-related surgery. Likewise, the level of ASCA IgG was also related to disease severity

## Data Availability Statement

The raw data supporting the conclusions of this article will be made available by the authors, without undue reservation.

## Ethics Statement

The studies involving human participants were reviewed and approved by the ethical committee of West China Hospital. Written informed consent for participation was not required for this study in accordance with the national legislation and the institutional requirements.

## Author Contributions

MJ and HZ contributed conception of the study. HZ contributed the design of the study. MJ and ZZ performed the statistical analysis and wrote the draft of the manuscript. KC, YD, LL, CM, RC, KH, XL wrote the draft of the manuscript. All the authors contributed to manuscript revision, read, and approved the submitted version. HZ was responsible for the final approval of the submitted version.

## Funding

This research was funded by the West China Hospital of Sichuan University 1.3.5 Excellence in Discipline Project (No. ZYJC18037).

## Conflict of Interest

The authors declare that the research was conducted in the absence of any commercial or financial relationships that could be construed as a potential conflict of interest.

## Publisher’s Note

All claims expressed in this article are solely those of the authors and do not necessarily represent those of their affiliated organizations, or those of the publisher, the editors and the reviewers. Any product that may be evaluated in this article, or claim that may be made by its manufacturer, is not guaranteed or endorsed by the publisher.

## References

[1] Lamb CA Kennedy NA Raine T Hendy PA Smith PJ Limdi JK. British Society of Gastroenterology Consensus Guidelines on the Management of Inflammatory Bowel Disease in Adults. Gut (2019) 68(Suppl 3):s1–106. doi: 10.1136/gutjnl-2019-318484 PMC6872448 31562236

[2] Clemente JC Manasson J Scher JU. The Role of the Gut Microbiome in Systemic Inflammatory Disease. Bmj (2018) 360:j5145. doi: 10.1136/bmj.j5145 29311119 PMC6889978

[3] Lichtenstein GR Loftus EV Isaacs KL Regueiro MD Gerson LB Sands BE. ACG Clinical Guideline: Management of Crohn’s Disease in Adults. Am J Gastroenterol (2018) 113(4):481–517. doi: 10.1038/ajg.2018.27 29610508

[4] Guindi M Riddell RH. Indeterminate Colitis. Springer London. J Clin Pathol (2004) 57(12):1233–44. doi: 10.1093/ecco-jcc/jjv063 PMC1770507 15563659

[5] Paul S Boschetti G Rinaudo-Gaujous M Moreau A Del Tedesco E Bonneau J. Association of Anti-Glycan Antibodies and Inflammatory Bowel Disease Course. J Crohns Colitis (2015) 9(6):445–51. doi: 10.1136/jcp.2003.015214 25895876

[6] Soubieres AA Poullis A. Emerging Role of Novel Biomarkers in the Diagnosis of Inflammatory Bowel Disease. World J Gastrointest Pharmacol Ther (2016) 7(1):41–50. doi: 10.4292/wjgpt.v7.i1.41 26855811 PMC4734953

[7] Dubinsky M Braun J. Diagnostic and Prognostic Microbial Biomarkers in Inflammatory Bowel Diseases. Gastroenterology (2015) 149(5):1265–1274.e1263. doi: 10.1053/j.gastro.2015.08.006 26284597 PMC5302020

[8] Barnes EL Burakoff R. New Biomarkers for Diagnosing Inflammatory Bowel Disease and Assessing Treatment Outcomes. Inflammation Bowel Dis (2016) 22(12):2956–65. doi: 10.1097/mib.0000000000000903 PMC5345848 27763951

[9] Kovács M. New Biomarkers in Pediatric Patients With Inflammatory Bowel Disease. World J Gastroenterol (2014) 20(17):4873. doi: 10.3748/wjg.v20.i17.4873 24803798 PMC4009518

[10] Miranda-Garcia P Chaparro M Gisbert JP. Correlation Between Gut Microbiome and Endoscopic Activity in Patients With Inflammatory Bowel Disease. Gastroenterol Hepatol (2016) 39(8):508–15. doi: 10.1016/j.gastrohep.2016.01.015 27020243

[11] Dotan I Fishman S Dgani Y Schwartz M Karban A Lerner A. Antibodies Against Laminaribioside and Chitobioside are Novel Serologic Markers in Crohn’s Disease. Gastroenterology (2006) 131(2):366–78. doi: 10.1053/j.gastro.2006.04.030 16890590

[12] Wang Z Zhu M Luo C Zhen Y Mu J Zhang W. High Level of Igg4 as a Biomarker for a New Subset of Inflammatory Bowel Disease. Sci Rep (2018) 8(1):10018. doi: 10.1038/s41598-018-28397-8 29968792 PMC6030091

[13] Alexander JJ Jacob A Chang A Quigg RJ Jarvis JN. Double Negative T Cells, a Potential Biomarker for Systemic Lupus Erythematosus. Precis Clin Med (2020) 3(1):34–43. doi: 10.1093/pcmedi/pbaa001 32257532 PMC7093895

[14] Maaser C Sturm A Vavricka SR Kucharzik T Fiorino G Annese V. ECCO-ESGAR Guideline for Diagnostic Assessment in Inflammatory Bowel Disease. J Crohns Colitis (2018) 13(2):144–64. doi: 10.1093/ecco-jcc/jjy113 30137275

[15] Limsrivilai J Shreiner AB Pongpaibul A Laohapand C Boonanuwat R Pausawasdi N. Meta-Analytic Bayesian Model for Differentiating Intestinal Tuberculosis From Crohn’s Disease. Am J Gastroenterol (2017) 112(3):415–27. doi: 10.1038/ajg.2016.529 PMC5551982 28045023

[16] Silverberg MS Satsangi J Ahmad T Arnott ID Bernstein CN Brant SR. Toward an Integrated Clinical, Molecular and Serological Classification of Inflammatory Bowel Disease: Report of a Working Party of the 2005 Montreal World Congress of Gastroenterology. Can J Gastroenterol (2005) 19 Suppl A(Suppl A):5A. doi: 10.1155/2005/269076 16151544

[17] Peyrinbiroulet L Standaertvitse A Branche J Chamaillard M. IBD Serological Panels: Facts and Perspectives. Inflammatory Bowel Dis (2007) 13(12):1561–6. doi: 10.1002/ibd.20226 17636565

[18] Malickova K Lakatos PL Bortlik M Komarek V Janatkova I Lukas M. Anticarbohydrate Antibodies as Markers of Inflammatory Bowel Disease in a Central European Cohort. Eur J Gastroenterol Hepatol (2010) 22(2):144–50. doi: 10.1097/MEG.0b013e32832f5c7e 19927001

[19] Kaul A Hutfless S Liu L Bayless TM Marohn MR Li X. Serum Anti-Glycan Antibody Biomarkers for Inflammatory Bowel Disease Diagnosis and Progression: A Systematic Review and Meta-Analysis. Inflamm Bowel Dis (2012) 18(10):1872–84. doi: 10.1002/ibd.22862 PMC3342398 22294465

[20] Ferrante M Henckaerts L Joossens M Pierik M Joossens S Dotan N. New Biomarkers in Inflammatory Bowel Disease are Associated With Complicated Disease Behaviour. Gut (2007) 56(10):1394–403. doi: 10.1136/gut.2006.108043 PMC2000264 17456509

[21] Reese GE Constantinides VA Simillis C Darzi AW Orchard TR Fazio VW. Diagnostic Precision of Anti-Saccharomyces Cerevisiae Antibodies and Perinuclear Antineutrophil Cytoplasmic Antibodies in Inflammatory Bowel Disease. Am J Gastroenterol (2006) 101(10):2410–22. doi: 10.1111/j.1572-0241.2006.00840.x 16952282

[22] Vasiliauskas EA Kam LY Karp LC Gaiennie J Yang H Targan SR. Marker Antibody Expression Stratifies Crohn’s Disease Into Immunologically Homogeneous Subgroups With Distinct Clinical Characteristics. Gut (2000) 47(4):487. doi: 10.1136/gut.47.4.487 10986208 PMC1728065

[23] Zhao XS Wang ZT Wu ZY Yin QH Zhong J Miao F. Differentiation of Crohn’s Disease From Intestinal Tuberculosis by Clinical and CT Enterographic Models. Inflammation Bowel Dis (2014) 20(5):916–25. doi: 10.1097/mib.0000000000000025 24694791

[24] Jung Y Hwangbo Y Yoon SM Koo HS Shin HD Shin JE. Predictive Factors for Differentiating Between Crohn’s Disease and Intestinal Tuberculosis in Koreans. Am J Gastroenterol (2016) 111(8):1156–64. doi: 10.1038/ajg.2016.212 27296940

[25] Singh SK Srivastava A Kumari N Poddar U Yachha SK Pandey CM. Differentiation Between Crohn Disease and Intestinal Tuberculosis in Children. J Pediatr Gastroenterol Nutr (2018) 66(1):e6–e11. doi: 10.1097/mpg.0000000000001625 28489674

[26] Yadav DP Madhusudhan KS Kedia S Sharma R Pratap Mouli V Bopanna S. Development and Validation of Visceral Fat Quantification as a Surrogate Marker for Differentiation of Crohn’s Disease and Intestinal Tuberculosis. J Gastroenterol Hepatol (2017) 32(2):420–6. doi: 10.1111/jgh.13535 27532624

[27] Zhang H Zeng Z Mukherjee A Shen B. Molecular Diagnosis and Classification of Inflammatory Bowel Disease. Expert Rev Mol Diagn (2018) 18(10):867–86. doi: 10.1080/14737159.2018.1516549 30152711

[28] Bae JH Park SH Ye BD Kim SO Cho YK Youn EJ. Development and Validation of a Novel Prediction Model for Differential Diagnosis Between Crohn’s Disease and Intestinal Tuberculosis. Inflammation Bowel Dis (2017) 23(9):1614–23. doi: 10.1097/mib.0000000000001162 28682807

